# Complete Genome Sequence of Rhodococcus qingshengii N9T-4

**DOI:** 10.1128/mra.00891-22

**Published:** 2022-11-08

**Authors:** Nobuhiro Obi, Ryota Moriuchi, Hideo Dohra, Kazuhide Kimbara, Masaki Shintani, Nobuyuki Yoshida

**Affiliations:** a Department of Engineering, Graduate School of Integrated Science and Technology, Shizuoka University, Hamamatsu, Shizuoka, Japan; b Research Institute of Green Science and Technology, Shizuoka University, Suruga-ku, Shizuoka, Japan; c Department of Science, Graduate School of Integrated Science and Technology, Shizuoka University, Suruga-ku, Shizuoka, Japan; d Japan Collection of Microorganisms, RIKEN BioResource Research Center, Tsukuba, Ibaraki, Japan; Wellesley College

## Abstract

Rhodococcus qingshengii N9T-4 can grow on media without added carbon sources. Here, we report the complete nucleotide sequence of the N9T-4 genome, consisting of a chromosome (6,439,972 bp), a linear plasmid (pN9T4-1 [565,206 bp]), and two circular plasmids (pN9T-4-2 [99,662 bp] and pN9T-4-3 [30,419 bp]).

## ANNOUNCEMENT

*Rhodococcus* strains can be isolated from various environments (1). Previously, bacterial strains that can grow on media without added carbon sources, belonging to the genera *Rhodococcus* and *Streptomyces*, were isolated (2). Rhodococcus qingshengii (formerly Rhodococcus erythropolis) N9T-4, which was isolated from the stocked crude oil in an oil stockpile in Japan, showed the best growth among our isolates, and CO_2_ is required for its growth (3). Although several genes that were upregulated under conditions without added carbon sources were identified (4, 5), complete genome sequencing is necessary to understand the carbon metabolism in N9T-4.

Total DNA of N9T-4 was extracted from 1 mL of overnight culture in Luria broth (6) using the NucleoBond high-molecular-weight (HMW) kit (Takara Bio, Shiga, Japan). The complete genome sequence of N9T-4 was determined using a combination of Pacific Biosciences (PacBio) Sequel and Illumina MiSeq sequencers. A PacBio 20-kb sequencing library was prepared using the SMRTbell template preparation kit v. 1.0 and sequenced with the Sequel platform (Macrogen, Inc., Seoul, South Korea). An Illumina sequencing library was constructed using the TruSeq Nano DNA library preparation kit and sequenced with the MiSeq platform. Sequel subreads were filtered (read lengths, ≥6,000 bp) using SeqKit v. 0.8.0 (7), and the resulting subreads were used for *de novo* assembly with Flye v. 2.8.3 (8) with the –plasmids option. Three circular contigs and one linear contig were obtained. Circularized contigs were rotated to start with the gene encoding the replication protein using Circlator v. 1.1.1 (9). Illumina paired-end (2 × 250-bp) reads were cleaned (read lengths, ≥150 bp; quality scores, ≥15) using Trimmomatic v. 0.39 (10), and the resulting high-quality short reads were used to correct Sequel errors using BWA-MEM v. 0.7.15 (11) and Pilon v. 1.23 (12). Mapping of short reads to all contigs indicated that there were no reads that mapped in pairs with the other contigs at either end of the linear contig, suggesting that the contig is a linear plasmid. Gene prediction and annotation were performed using DFAST v. 1.2.15 (13) by running GeneMarkS2 v. 1.14_1.25 (14), RNAmmer v. 1.2 (15), and tRNAscan-SE v. 2.0.5 (16). Default parameters were used for all software unless otherwise specified. Information on the PacBio and Illumina reads is summarized in Table 1.

**TABLE 1:** Summary of reads generated by Illumina and PacBio sequencing

Parameter	Data for sequencing with:	
	Illumina MiSeq	PacBio Sequel
Raw reads		
No. of reads	1,558,816	73,378
Total size (bp)	390,155,166	779,091,421
*N*_50_ (bp)		13,738
Filtered reads		
No. of reads	1,385,380	54,401
Total size (bp)	339,591,845	723,937,732
*N*_50_ (bp)		14,230
Mean coverage (×)^*a*^	48	101
Accession no.	DRR402011	DRR402012

a Total size of filtered reads (bp)/genome size of N9T-4 (bp).

The genome of N9T-4 consists of a circular chromosome (6,439,972 bp, with a G+C content of 62%), a linear plasmid (pN9T4-1 [565,206 bp], with a G+C content of 62%), and two circular plasmids (pN9T4-2 [99,662 bp] and pN9T4-3 [30,419 bp], with G+C contents of 62% and 63%, respectively). The genome has 6,738 coding sequences, 5 sets of rRNA genes, and 58 tRNA genes. Average nucleotide identity (ANI) analysis was performed with calcANI.pl (https://github.com/Computational-conSequences/SequenceTools) with the ANIb method using 586 genome sequences of *Rhodococcus* strains in the NCBI RefSeq database (as of 30 March 2022). The ANI analysis of N9T-4 showed high ANI values (>95%) with respect to the type strains R. qingshengii djl-6 (98.49%) (accession number CP096563) (17) and R. erythropolis JCM 3201 (accession number GCA_003990875) (18) (95.27%), suggesting that N9T-4 could be recognized as R. qingshengii.

### Data availability.

The nucleotide sequences of the N9T-4 chromosome and plasmids have been deposited in the DDBJ/EMBL/GenBank databases under accession numbers AP026691 (chromosome) and AP026692, AP026693, and AP026694 (plasmids pN9T4-1, pN9T4-2, and pN9T4-3, respectively). The raw data are available under DRA accession number DRA014768 for Illumina (accession number DRR402011) and PacBio (accession number DRR402012) sequencing. All project data are available under BioProject accession number PRJDB13588.

## References

[1] Yoshida N. 2019. Oligotrophic growth of *Rhodococcus*, p 87–101. *In* Biology of *Rhodococcus*, 2nd ed. Springer Nature Switzerland AG, Cham, Switzerland.

[2] Yoshida N, Ohhata N, Yoshino Y, Katsuragi T, Tani Y, Takagi H. 2007. Screening of carbon dioxide-requiring extreme oligotrophs from soil. Biosci Biotechnol Biochem 71:2830–2832. doi:10.1271/bbb.70042. 17986768

[3] Ohhata N, Yoshida N, Egami H, Katsuragi T, Tani Y, Takagi H. 2007. An extremely oligotrophic bacterium, *Rhodococcus erythropolis* N9T-4, isolated from crude oil. J Bacteriol 189:6824–6831. doi:10.1128/JB.00872-07. 17675378 PMC2045210

[4] Yoshida N, Hayasaki T, Takagi H. 2011. Gene expression analysis of methylotrophic oxidoreductases involved in the oligotrophic growth of *Rhodococcus erythropolis* N9T-4. Biosci Biotechnol Biochem 75:123–127. doi:10.1271/bbb.100700. 21228466

[5] Ikeda Y, Kishimoto M, Shintani M, Yoshida N. 2022. Oligotrophic gene expression in *Rhodococcus erythropolis* N9T-4 under various nutrient conditions. Microorganisms 10:1725. doi:10.3390/microorganisms10091725. 36144327 PMC9502362

[6] Sambrook J, Russell D. 2001. Molecular cloning: a laboratory manual, 3rd ed. Cold Spring Harbor Laboratory, Cold Spring Harbor, NY.

[7] Shen W, Le S, Li Y, Hu F. 2016. SeqKit: a cross-platform and ultrafast toolkit for FASTA/Q file manipulation. PLoS One 11:e0163962. doi:10.1371/journal.pone.0163962. 27706213 PMC5051824

[8] Kolmogorov M, Yuan J, Lin Y, Pevzner PA. 2019. Assembly of long, error-prone reads using repeat graphs. Nat Biotechnol 37:540–546. doi:10.1038/s41587-019-0072-8. 30936562

[9] Hunt M, Silva ND, Otto TD, Parkhill J, Keane JA, Harris SR. 2015. Circlator: automated circularization of genome assemblies using long sequencing reads. Genome Biol 16:294. doi:10.1186/s13059-015-0849-0. 26714481 PMC4699355

[10] Bolger AM, Lohse M, Usadel B. 2014. Trimmomatic: a flexible trimmer for Illumina sequence data. Bioinformatics 30:2114–2120. doi:10.1093/bioinformatics/btu170. 24695404 PMC4103590

[11] Li H. 2013. Aligning sequence reads, clone sequences and assembly contigs with BWA-MEM. arXiv 1303.3997. https://arxiv.org/pdf/1303.3997.pdf.

[12] Walker BJ, Abeel T, Shea T, Priest M, Abouelliel A, Sakthikumar S, Cuomo CA, Zeng Q, Wortman J, Young SK, Earl AM. 2014. Pilon: an integrated tool for comprehensive microbial variant detection and genome assembly improvement. PLoS One 9:e112963. doi:10.1371/journal.pone.0112963. 25409509 PMC4237348

[13] Tanizawa Y, Fujisawa T, Nakamura Y. 2018. DFAST: a flexible prokaryotic genome annotation pipeline for faster genome publication. Bioinformatics 34:1037–1039. doi:10.1093/bioinformatics/btx713. 29106469 PMC5860143

[14] Lomsadze A, Gemayel K, Tang S, Borodovsky M. 2018. Modeling leaderless transcription and atypical genes results in more accurate gene prediction in prokaryotes. Genome Res 28:1079–1089. doi:10.1101/gr.230615.117. 29773659 PMC6028130

[15] Lagesen K, Hallin P, Rødland EA, Staerfeldt H-H, Rognes T, Ussery DW. 2007. RNAmmer: consistent and rapid annotation of ribosomal RNA genes. Nucleic Acids Res 35:3100–3108. doi:10.1093/nar/gkm160. 17452365 PMC1888812

[16] Chan PP, Lin BY, Mak AJ, Lowe TM. 2021. tRNAscan-SE 2.0: improved detection and functional classification of transfer RNA genes. Nucleic Acids Res 49:9077–9096. doi:10.1093/nar/gkab688. 34417604 PMC8450103

[17] Xu J-L, He J, Wang Z-C, Wang K, Li W-J, Tang S-K, Li S-P. 2007. *Rhodococcus qingshengii* sp. nov., a carbendazim-degrading bacterium. Int J Syst Evol Microbiol 57:2754–2757. doi:10.1099/ijs.0.65095-0. 18048720

[18] Goodfellow M, Alderson G. 1977. The actinomycete-genus *Rhodococcus*: a home for the “rhodochrous” complex. J Gen Microbiol 100:99–122. doi:10.1099/00221287-100-1-99. 874450

